# A Molecular Score by Quantitative PCR as a New Prognostic Tool at Diagnosis for Chronic Lymphocytic Leukemia Patients

**DOI:** 10.1371/journal.pone.0012780

**Published:** 2010-09-16

**Authors:** Basile Stamatopoulos, Nathalie Meuleman, Cécile De Bruyn, Karlien Pieters, Géraldine Anthoine, Philippe Mineur, Dominique Bron, Laurence Lagneaux

**Affiliations:** 1 Laboratory of Experimental Hematology, Faculty of Medicine, Institut Jules Bordet, Université Libre de Bruxelles (ULB), Brussels, Belgium; 2 Department of Intensive Care and Thoracic Oncology, Institut Jules Bordet, Université Libre de Bruxelles (ULB), Brussels, Belgium; 3 Department of Hemato-Oncology, Grand Hôpital de Charleroi, Gilly, Belgium; University of Barcelona, Spain

## Abstract

**Background:**

Several markers have been proposed to predict the outcome of chronic lymphocytic leukemia (CLL) patients. However, discordances exist between the current prognostic factors, indicating that none of these factors are totally perfect.

**Methodology/Principal Findings:**

Here, we compared the prognostic power of new RNA-based markers in order to construct a quantitative PCR (qPCR) score composed of the most powerful factors. ZAP70, LPL, CLLU1, microRNA-29c and microRNA-223 were measured by real time PCR in a cohort of 170 patients with a median follow-up of 64 months (range3-330). For each patient, cells were obtained at diagnosis and RNA was extracted from purified CD19 cells. The best markers were included in a qPCR score, which was thereafter compared to each individual factor. Statistical analysis showed that all five RNA-based markers can predict treatment-free survival (TFS), but only ZAP70, LPL and microRNA-29c could significantly predict overall survival (OS). These three markers were thus included in a simple qPCR score that was able to significantly predict TFS and OS by dividing patients into three groups (0/3, 1-2/3 and 3/3). Median TFS were >210, 61 and 24 months (P<0.0001) and median OS were >330, 242 and 137 months (P<0.0001), respectively. Interestingly, TFS results were also confirmed in Binet stage A patients (P<0.0001). When compared to other classical factors, this score displays the highest univariate Cox hazard ratio (TFS: HR = 9.45 and OS: HR = 13.88) but also provides additional prognostic information.

**Conclusions:**

In our hands, this score is the most powerful tool for CLL risk stratification at the time of diagnosis.

## Introduction

Within the past decade, several markers have been proposed to predict the outcome of chronic lymphocytic leukemia (CLL) patients [1] . This disease is characterized by an accumulation of B cells and is greatly heterogeneous in terms of clinical course. Half of the patients display an indolent and stable disease, whereas the other half displays a very aggressive disease with poor outcome [2]. While the “old” parameters such as clinical stage (Rai [3] or Binet [4]) are unable to prospectively distinguish early-stage CLL that progresses rapidly to aggressive disease from disease destined to remain in an early stage for an extended time, the “new” parameters described since 1999 have considerably improved disease risk classification. One of the most important prognostic molecular factors is the mutational status of the immunoglobulin heavy chain region (IgVH). Indeed, patients presenting with an unmutated IgVH had a worse outcome than patients with mutated IgVH, who had a good prognosis [5], [6]. However, this analysis is laborious and costly as well as inaccessible for most clinical laboratories. Therefore, in an effort to simplify this procedure, several attempts have been made to replace this analysis with an efficient surrogate marker. Numerous genes were suggested based on gene expression profiles comparing IgVH mutated and unmutated patients [7], [8]. Of these, ZAP70 (zeta-associated protein 70) and LPL (lipoprotein lipase) expression was associated with IgVH status and with poor outcome, and their prognostic significance has now been confirmed by several studies (reviewed in [1]). Furthermore, accurate and standardized methods to measure these two markers by quantitative real-time PCR (qPCR) have been described [9], [10]. Other RNA-based prognostic factors such as CLLU1 [11], microRNA-29c (or miR-29c) and microRNA-223 (or miR-223) [12] have also been proposed. However, several discordances exist between these RNA-based markers, indicating that none of these prognostic factors is totally perfect to predict TFS or OS. In addition, the use of only one factor could lead to the misclassification of the patient, whereas a combination of factors could reduce this risk. Managing the treatment course of CLL patients cannot be planned without taking their prognosis into account. Therefore, in the present study we aimed to construct a molecular qPCR score composed of the best prognostic factors among the five above-mentioned RNA-based markers in order to improve CLL patient risk stratification at diagnosis. Furthermore, because the majority of patients (70–80%) are diagnosed at an early stage and an efficient prognostic factor should be able to identify those patients with a higher risk of progressive disease at the time of diagnosis, we also evaluated the developed score in newly diagnosed stage A CLL patients. Finally, we compared this score to each individual marker and also to the currently used prognostic factors [IgVH mutational status, Binet stage, β2-microglobulin (β2-M), soluble CD23 (sCD23), lymphocyte doubling time (LDT), CD38 molecule (CD38) and cytogenetic abnormalities]. The additional power of this qPCR score was also tested in poor or good prognosis subgroups defined by other prognostic markers.

## Results

### Patient characteristics

The main characteristics of this 170 patient cohort are shown in Table 1. The male to female ratio was 1.7 and the median age at diagnosis was 64 years (range 34-89), with 46% of patients aged more than 65 years and 30% aged more than 70 years. Binet stage distribution was 73, 18 and 9%, respectively, for Binet stage A, B and C. The median TFS of this cohort was 76 months (range, 0.03–210 months), whereas the median OS was 242 months (range, 0.5–330 months). The median follow-up was 64 months (range 3–330). Of the total 170 patients, 84 patients (49%) received a treatment, whereas only 24 patients (14%) died. Of the 124 Binet stage A patients, 43 patients (35%) received a treatment, whereas only 8 patients (6%) died. Of the 46 Binet stage B/C, 41 patients (89%) received a treatment, whereas 16 patients (35%) died Complete prognostic data were not available for all patients due to a lack of biological material (Table 1). For Binet stages, ZAP70, LPL, CLLU1, microRNA-29c and microRNA-223 expression, clinical data were available for all of the 170 patients included in this study. Incomplete data were available for IgVH mutational status (77% of the patients), CD38 expression (95%), LDT (73%), β2-M (94%), sCD23 (90%) and cytogenetic abnormalities (68%).

**Table 1:** Patient characteristics and TFS/OS analysis.

			Treatment-free survival						Overall survival					
			Kaplan-Meier curves			Univariate Cox regression			Kaplan-Meier curves			Univariate Cox regression		
	n	%	median TFS (months)	*P*	*χ^2^(1)*	*HR*	*Cl 95%*	*P*	median OS (months)	*P*	*χ^2^(1)*	*HR*	*Cl 95%*	*P*
**Patients**	170													
** Male**	106	*62*	62.6	0.3453	0.89	1.24	0.79–1.93	0.3465	241.9	0.9015	0.02	1.05	0.46–2.42	0.9015
** Female**	64	*38*	87.2						225.1					
**Binet**	170													
** Stage A**	124	*73*	94.4	*P<0.0001*	41.35	5.57	3.19–9.73	*P<0.0001*	>330	0.0006	14.72	5.68	2.18–14.79	0.0004
** Stage B**	31	*18*	26.6						241.9					
** Stage C**	15	*9*	24.0						106.2					
**Mutational status** a	131													
** IgVH - Unmutated**	60	*46*	26.6	*P<0.0001*	33.09	4.12	2.45–6.92	*P<0.0001*	183.0	0.0011	10.70	4.61	1.69–12.56	0.0028
** IgVH - Mutated**	71	*54*	129.5						>330					
**ZAP-70** b	170													
** >134.9 (positive)**	81	*48*	25.2	*P<0.0001*	51.35	5.26	3.20–8.67	*P<0.0001*	152.5	*P<0.0001*	17.98	7.41	2.52–21.84	0.0003
** <134.9 (negative)**	89	*52*	157.0						>330					
**LPL** b	170													
** >15.12 (positive)**	65	*38*	26.6	*P<0.0001*	28.69	3.09	2.00–4.78	*P<0.0001*	225.1	0.0240	5.10	2.51	1.10–5.75	0.0292
** <15.12 (negative)**	105	*62*	129.5						241.9					
**CLLU1** b	170													
** >14.42 (positive)**	87	*51*	52.4	0.0292	4.75	1.62	1.05–2.50	0.0309	225.1	0.6726	0.18	1.19	0.53–2.69	0.6730
** <14.42 (negative)**	83	*49*	126.0						>330					
**microRNA-29c** b	170													
** <4.64 (negative)**	70	*41*	37.0	0.0002	13.73	2.22	1.44–3.42	0.0003	183.0	0.0015	10.11	4.88	1.66–14.35	0.0040
** >4.64 (positive)**	100	*59*	93.7						>330					
**microRNA-223** b	170													
** <138 (negative)**	100	*59*	42.2	0.0004	12.69	2.42	1.46–4.00	0.0006	241.9	0.8195	0.05	1.11	0.46–2.70	0.8196
** >138 (positive)**	70	*41*	126.0						225.1					
**CD38** b	161													
** >7% (positive)**	74	*46*	35.5	*P<0.0001*	18.96	2.68	1.69–4.25	*P<0.0001*	241.9	0.0036	8.47	3.76	1.45–9.77	0.0066
** <7% (negative)**	87	*54*	129.5						>330					
**LDT**	124													
** < 1year**	31	*25*	18.7	*P<0.0001*	39.51	4.35	2.64–7.18	*P<0.0001*	137.2	0.0035	8.54	3.29	1.41–7.66	0.0057
** > 1year**	93	*75*	107.2						241.9					
**Beta-2-microglubulin** b	159													
**>2.77 µg/ml (positive)**	73	*46*	40.0	0.0005	12.09	2.20	1.40–3.48	0.0007	225.1	0.0188	5.52	3.08	1.15–8.26	0.0257
**<2.77 µg/ml (negative)**	86	*54*	107.2						>330					
**soluble CD23** b	153													
** >120 U (positive)**	80	52	28.6	*P<0.0001*	25.57	3.49	2.08–5.84	*P<0.0001*	225.1	0.0069	7.29	4.59	1.36–15.44	0.0139
** <120 U (negative)**	73	48	178.4						>330					
**Cytogenetic abnormalities** c	115													
** del(17p), (11q), (6q), +12, complex**	51	44	37.0	0.0029	8.84	2.29	1.31–4.01	0.0038	225.1	0.0287	4.78	3.24	1.06–9.86	0.0386
** normal,del(13q), other**	64	56	88.5						>330					
**Quantitative PCR score**	170													
** 0/3**	56	33	>210	*P<0.0001*	48.90	9.45	4.85–18.44	*P<0.0001*	>330	*P<0.0001*	18.63	13.88	3.61–53.41	0.0001
** 1**–**2/3**	80	47	60.1						241.9					
** 3/3**	34	20	24.0						137.2					

a Mutational status is based on a 98% cut-off value.

b The cut-off determined using ROC curve analysis maximising the concordance with the IgVH status.

c Among patients with unfavorable cytogenetic abnormalities (n = 51), we found 9 patients with a del(17p), 13 with a del(11q), 10 with del(6q), and 18 with a trisomy-12. Furthermore, 1patient presents a complex karyotype associated with poor prognosis. Among patients with favorable cytogenetic abnormalities (n = 64), we found 31 patients with del(13q) and 3 patients with other(s) abnormalities. 30 patients had a normal karyotype.

### Prognostic power of individual markers: TFS and OS analysis

**Figure 1:**
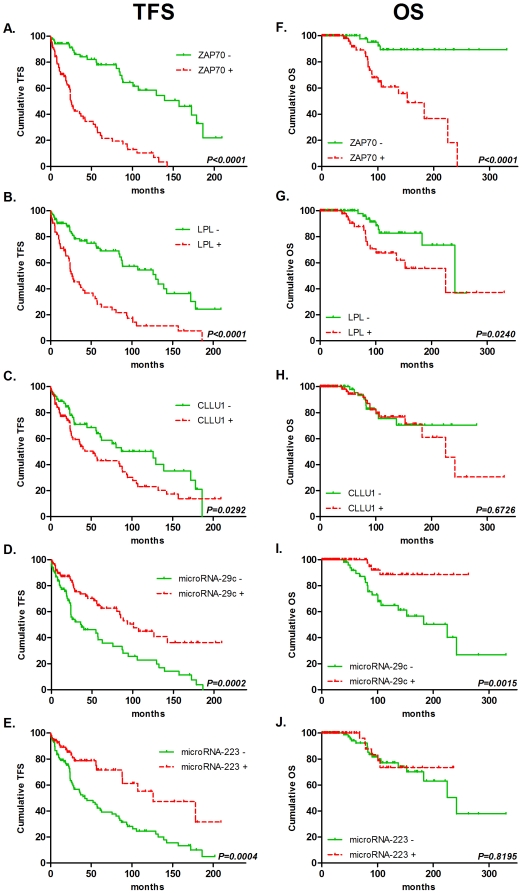
Kaplan-Meier survival curves for TFS/OS of RNA-based markers. TFS and OS were plotted using Kaplan-Meier methods, and curves were compared with the log-rank test (n = 170). ZAP70 (A and F), LPL (B and G), CLLU1 (C and H), microRNA-29c (D and I) and microRNA-223 (E and J). All cut-offs were determined using ROC curve analysis. Further information can be found in Table 1.

All cut-offs were calculated using ROC curve analysis to maximize the concordance with the IgVH mutational status and are provided in Table 1. Although these cut-offs were not always optimized for TFS/OS prediction, they were sufficient to observe the following statistical differences: among our 170 patients, all RNA-based tested markers (ZAP70, LPL, CLLU1, microRNA-29c and microRNA-223) were able to significantly predict TFS, whereas only ZAP70, LPL and microRNA-29c were significant OS predictors as calculated by Kaplan-Meier curves (with log-rank test) and univariate Cox regression after dichotomisation of the data (Table 1 and Figure 1). The prognostic power of other classical factors was also evaluated and is stated in Table 1.

### Construction and prognostic evaluation of the qPCR score

**Figure 2:**
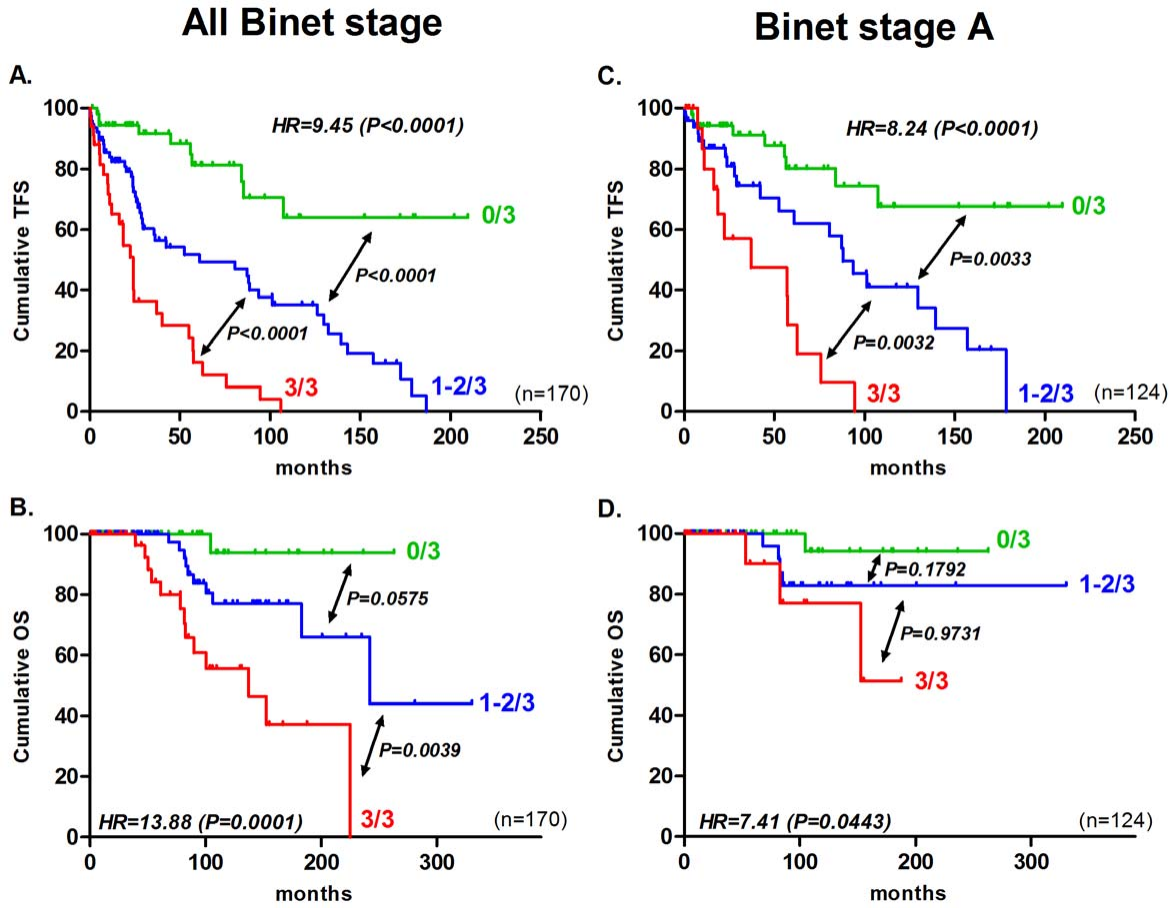
qPCR combining ZAP70, LPL and microRNA-29c stratifies CLL patients in terms of TFS and OS. TFS and OS, according to our qPCR score, were plotted with Kaplan-Meier methods for all Binet stages (A and B) and only Binet stage A (C and D). Curves were compared with the log-rank test (all Binet stages, n = 170; Binet stage A, n = 124). The hazard ratio (HR) was calculated with univariate Cox regression. Further information can be found in Table 1.

The aim of this study was to construct a score using a unique and standardized technique in order to refine CLL prognosis methods. To this end, we constructed a simple and easy qPCR score by including the best individual prognostic factors, namely ZAP70, LPL and microRNA-29c. Indeed, only these three markers could significantly predict both TFS and OS. Furthermore, they are measurable by qPCR and thus no other techniques, such as flow cytometry or FISH analysis, are needed. This qPCR score varied from 0 to 3 according to the number of unfavorable factors (i.e., low expression of miR-29c and high expression of ZAP70 or LPL). The presence of a poor prognostic marker corresponds to an increase of 1 unit in the final qPCR score. We gave the same weight to all three factors. According to this qPCR score, the patients were thus stratified into three groups: 0/3 (all factors classified the patients in the favourable prognostic group), 3/3 (all factors classified the patients in the unfavourable prognostic group) and finally 1-2/3 (at least one factor classified patients in an unfavourable prognostic group). The hazard ratio (HR) of these three groups (named 0, 0.5, and 1) was calculated by univariate Cox analysis. In other words, HR represents the hazard ratio between group 0/3 and 3/3 taking into account the intermediate group such that 0/3<1–2/3<3/3. Patients with a score of 0/3, 1–2/3, and 3/3 had a median TFS of >210, 60 and 24 months, respectively (*HR = 9.5, P<0.0001*) and a median OS of >330, 242 and 137 months, respectively (*HR = 13.9, P = 0.0001*) (Figure 2). Finally, in Binet stage A patients (n = 124), this score remained relevant and significant for TFS. Patients with a score of 0/3, 1–2/3, and 3/3 had a median TFS of >210, 88 and 37 months, respectively (*HR = 8.2*, *P<0.0001*). For the prediction of OS, the hazard ratio remains significant (*HR = 7.4*, *P = 0.0443*), but the median OS for each group could not be reached and were, thus, not calculable due to a limited number of events (n = 8) (Figure 2). When only Binet Stage B/C were considered (n = 46), our score displays a significant HR for TFS (*HR = 5.5, P = 0.0095*) and OS (*HR = 11.2, P = 0.0265*).

### Additional impact of the qPCR score on other prognostic factors

**Figure 3:**
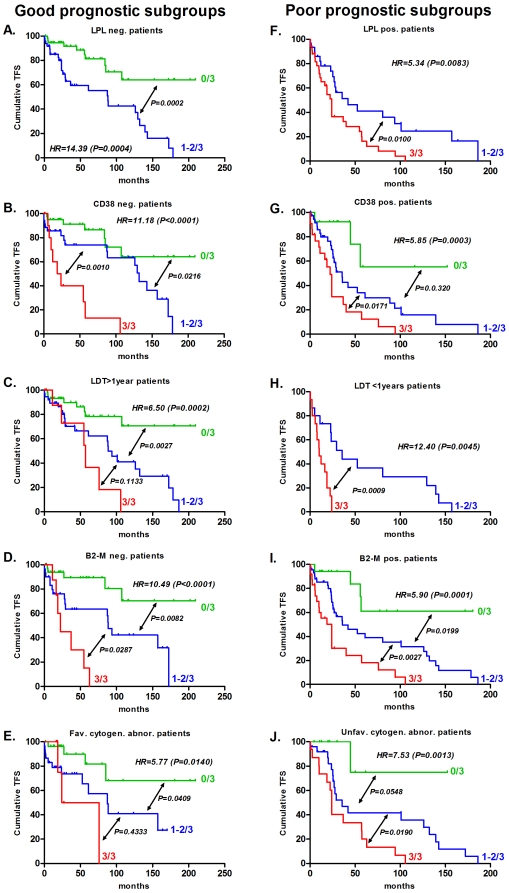
qPCR can fine tune prognosis in good or poor prognostic subgroups. The qPCR score was applied to different subgroups (previously divided by other prognostic factors). TFS curves are shown for LPL (A, F), CD38 (B, G), LDT (C, H), β2-M (D, I), and cytogenetic abnormalities (E, J). The median TFS and patient numbers in the different groups are provided in Table 2. Groups of less than two patients are not represented in this figure.

**Figure 4:**
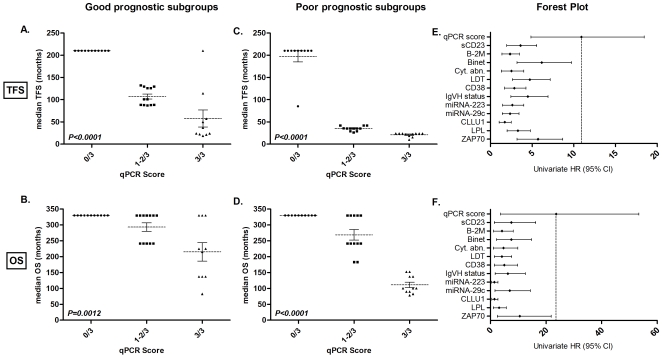
Supplementary prognostic value of the qPCR score. Each prognostic factor was used to divide the patient cohort in two different prognostic subgroups according to prognostic factors reported in Table 1. The qPCR score was then applied to all poor prognosis subgroups and good prognosis subgroups. (A) and (B) show the median TFS and median OS of the good prognosis subgroups, whereas (C) and (D) show the median TFS and median OS of the poor prognostic subgroups. The dotted line and error bar represent the mean and the SEM, respectively. Statistical differences were assessed using the Kruskal-Wallis test. Parts (E) and (F) show forest plots comparing the univariate Cox HR of the qPCR score with other prognostic factors for TFS and OS prediction, respectively. The hazard ratio (HR) of all variables was calculated by univariate Cox analysis and plotted with the 95% Cl on this forest plot. More details can be found in Table 2.

To evaluate the additional impact of our qPCR score, we applied this score to good and poor prognostic subgroups defined by the 12 prognostic factors stated in Table 2. We, thus, obtained 24 subgroups (12 of good and 12 of poor prognosis). We divided each of these subgroups according to the qPCR score, and we calculated the median TFS and the median OS for all of the subgroups generated. The univariate Cox HR was calculated as described above. Interestingly, we observed that this qPCR score allowed the identification of patients with a higher median TFS or OS in poor prognostic subgroups and of patients with lower TFS or OS in good prognosis subgroups (Table 2). Some examples of this additional power for TFS were illustrated by Kaplan-Meier curves (performed in poor/good prognostic subgroups) (Figure 3). The qPCR score was able to divide patients into subgroups previously classified by RNA-based, flow cytometric, proliferation, serum and cytogenetic prognostic markers. Independently from the chosen subgroups, all patients with any poor or good prognostic factors but with a score of 0/3 had a median TFS >210 months (except Binet Stage B/C patients) and a median OS >330 months. It should be noted that all HR_0/3<1–2/3<3/3_ were significant for TFS prediction, but not for OS, probably because of the limited number of events per subgroup. Therefore, we analysed the impact of our qPCR score more globally. Each TFS and OS for all of the poor prognosis subgroups and all of the good prognosis subgroups were plotted together (Figure 4A–D). A clear trend indicating a reduction of median TFS and OS was observed, and a Kruskal-Wallis test demonstrated that this qPCR score could find at least two distinct subgroups with significantly different TFS [in good (*P<0.0001*) and poor (*P<0.0001*) subgroups] or OS [in good (*P = 0.0012*) and poor (*P<0.0001*) subgroups]. Figure 4E–F forest plots also show qPCR strength compared to the other analysed prognostic markers.

**Table 2:** Additional power of the qPCR score on different prognostic subgroups.

		Treatment-free survival									Overall survival								
		qPCR score median TFS						Univariate Cox regression			qPCR score median OS						Univariate Cox regression		
	*n*	0/3	*n*	1–2/3	*n*	3/3	*n*	HR	95% Cl	P	0/3	*n*	1–2/3	*n*	3/3	*n*	HR	95% Cl	P
**Binet Stage**	***170***																		
** Stage A**	***##***	>210	54	88.1	52	37.0	18	8.24	3.39–20.00	*P<0.0001*	*>330*	*54*	*>330*	*52*	*>330*	*18*	7.41	1.05–52.21	0.0443
** Stage B-C**	***46***	85.0	*2*	26.6	*28*	15.5	*16*	4.98	1.48–16.73	0.0095	>330	*2*	241.9	*28*	100.5	*16*	11.17	1.33–94.08	0.0265
**Mutational status** a	*131*																		
** IgVH - Unmutated**	*60*	>210	*2*	29.0	*30*	22.3	*28*	3.83	1.23–11.95	0.0205	>330	*2*	183.0	*30*	100.5	*28*	7.71	0.81–73.25	0.7529
** IgVH - Mutated**	*71*	>210	*38*	101.0	*32*	>210	*1*	7.39	1.41–38.84	0.0181	>330	*38*	>330	*32*	>330	*1*	9.59	0.12–767.95	0.3122
**ZAP-70** b	*170*																		
** >134.9 (positive)**	*81*	-	*0*	29.0	*47*	24.0	*34*	3.03	1.03–8.88	0.0430	-	*0*	183.0	*47*	137.2	*34*	4.20	0.60–29.19	0.1466
** <134.9 (negative)**	*89*	>210	*56*	129.5	*33*	-	*0*	6.03	1.25–29.08	0.0251	>330	*56*	>330	*33*	-	*0*	8.72	0.09–806.91	0.3486
**LPL** b	*170*																		
** >15.12 (positive)**	*65*	-	*0*	42.2	*31*	24.0	*34*	5.34	1.54–18.51	0.0083	-	*0*	>330	*31*	137.2	*34*	15.66	2.32–105–91	0.0048
** <15.12 (negative)**	*105*	>210	*56*	88.1	*49*	-	*0*	14.39	3.34–62.09	0.0004	>330	*56*	241.8	*49*	-	*0*	18.43	2.47–137.43	0.0045
**CLLU1** b	*170*																		
** >14.42 (positive)**	*87*	>210	*22*	35.5	*42*	24.1	*23*	7.25	3.09–16.97	*P<0.0001*	>330	*22*	241.9	*42*	152.5	*23*	15.66	2.32–105.91	0.0048
** <14.42 (negative)**	*83*	>210	*34*	126.0	*38*	18.7	*11*	11.31	3.74–34.21	*P<0.0001*	>330	*34*	>330	*38*	82.6	*11*	18.43	2.47–137.43	0.0045
**microRNA-29c** b	*170*																		
** <4.64 (negative)**	*70*	-	*0*	129.5	*36*	24.0	*34*	14.51	3.75–56.10	0.0001	-	*56*	241.9	*44*	137.2	*0*	9.40	1.30–67.71	0.0261
** >4.64 (positive)**	*100*	>210	*56*	42.2	*44*	-	*0*	19.86	4.58–86.10	*P<0.0001*	>330	*0*	>330	*36*	-	*34*	15.21	0.16–1414.51	0.2392
**microRNA-223** b	*170*																		
** <138 (negative)**	*100*	>210	*20*	42.2	*49*	24.0	*31*	7.93	3.18–17.55	*P<0.0001*	>330	*49*	241.9	*31*	137.2	*3*	25.61	3.80–172.63	0.0009
** >138 (positive)**	*70*	>210	*36*	126.0	*31*	114.2	*3*	7.47	1.50–37.11	0.0139	>330	*20*	>330	*36*	78.4	*31*	30.19	1.15–794.10	0.0411
**CD38** b	*161*																		
** >7% (positive)**	*74*	>210	*13*	35.5	*39*	22.3	*22*	5.85	2.24–15.27	0.0003	>330	*13*	>330	*39*	89.9	*22*	11.77	1.53–9067	0.0180
** <7% (negative)**	*87*	>210	*41*	132.3	*35*	21.3	*11*	11.18	3.86–32.39	*P<0.0001*	>330	*41*	241.9	*35*	225.1	*11*	24.26	1.95–302–40	0.0132
**LDT**	*124*																		
** < 1year**	*31*	>210	*1*	35.5	*15*	10.1	*15*	12.40	2.19–70.33	0.0045	>330	*1*	>330	*15*	82.6	*15*	21.31	1.64–275.52	0.0192
** > 1year**	*93*	>210	*45*	88.5	*38*	57.0	*10*	6.50	2.39–17.64	0.0002	>330	*45*	241.9	*38*	225.1	*10*	7.13	0.98–51.66	0.0519
**Beta-2-microglubulin** b	*159*																		
**>2.77 µg (positive)**	*73*	>210	*17*	36.0	*45*	24.0	*24*	5.90	2.43–14.34	0.0001	>330	*17*	241.9	*45*	100.5	*24*	12.80	2.33–70.38	0.0034
**<2.77 µg (negative)**	*86*	>210	*34*	88.5	*30*	22.3	*9*	10.49	3.26–33.79	*P<0.0001*	>330	*34*	>330	*30*	>330	*9*	9.99	0.70–142.29	0.0894
**soluble CD23** b	*153*																		
** >120 U (positive)**	*73*	>210	*7*	35.5	*40*	18.7	*26*	5.24	2.11–13.03	0.0004	>330	*7*	241.9	*40*	152.5	*26*	6.48	1.33–31.65	0.0208
** <120 U (negative)**	*80*	>210	*42*	101	*32*	24.0	*6*	7.36	1.61–33.62	0.0100	>330	*42*	>330	*32*	137.2	*6*	47.58	1.13–2009.23	0.0431
**Cytogenetic abnormalities** c	*115*																		
** del(17p), (11q), (6q), +12, complex**	*51*	>210	*10*	35.5	*25*	24.0	*16*	7.53	2.19–25.76	0.0013	>330	*10*	241.9	*25*	89.9	*16*	35.05	2.76–445.79	0.0061
** normal,del(13q), other**	*64*	>210	*28*	87.2	*30*	50.0	*6*	5.77	1.43–23.34	0.0140	>330	*28*	>330	*30*	215.5	*6*	8.67	0.47–158.47	0.4450

a Mutational status is based on a 98% cut-off value.

b The cut-off determined using ROC curve analysis maximising the concordance with the IgVH status

c Among patients with unfavorable cytogenetic abnormalities (n = 51), we found 9 patients with a del(17p), 13 with a del(11q), 10 with del(6q), and 18 with a trisomy-12. Furthermore, 1patient presents a complex karyotype associated with poor prognosis. Among patients with favorable cytogenetic abnormalities (n = 64), we found 31 patients with del(13q) and 3 patients with other(s) abnormalities. 30 patients had a normal karyotype.

## Discussion

The aim of this study was to propose a simple tool in order to asses CLL prognosis at the time of diagnosis. In recent years, an increasing number of studies have proposed scores or indexes that are gradually replacing the use of monoparametric prognostic markers [18]–[20]. Indeed, clinical stages such as Binet [4] or Rai [3] suffer from great prognostic heterogeneity. Furthermore, with the widespread of blood analysis 70–80% of CLL patients are diagnosed at an early stage, limiting the utility of this easy prognostic tool. New individual markers including proliferation, surface, molecular and cytogenetic markers have been proposed. However, several discordances exist between the current prognostic factors, indicating that none of these factors are optimal, and each of these prognostic factors alone has limited utility in predicting TFS or OS. Therefore, a better risk stratification for an individualized and tailored follow-up of the patient is needed. In 2006, Zuchetto et al. constructed a scoring system based on six surface expression molecules [19]. In 2007, Wierda et al. proposed a prognostic index based on a large number of patients that was composed of six variables, including clinical parameters and proliferation markers such as beta-2-microglobulin [18]. These two studies both used OS as the end point but did not investigate TFS. In 2010, Kienle et al. proposed a prognostic model based on gene expression markers and concluded that this model could not replace the established prognostic factors, but can improve TFS and OS prediction [20]. All of these classifiers have some degree of complexity because of the larger number of parameters and are, therefore, not always practical for physicians. In our previous work, we demonstrated that microRNAs (miR-29c and miR-223) could also add prognostic information to classical factors (ZAP70 and LPL) in a qPCR score that is able to classify patients into five (for TFS) or three (for OS) groups with decreasing median TFS or OS [12]. This score already used less parameters than other scoring systems (with the advantage of using a single technique) but had a weak prognostic power during the two first years after diagnosis.

In the present work, we wanted to construct an easy tool for clinicians to use with a minimum number of parameters and without difficult calculations or interpretations, as was the case for Binet stages. To this end, we first investigated RNA-based markers by univariate Cox regression for TFS and OS prediction. In a second step, we only included in our qPCR score markers, which were significant TFS and OS univariate predictors (ZAP70, LPL and microRNA-29c). ZAP70 and LPL have been well documented in the literature, and their prognostic power is no longer contested [1]. The prognostic value of microRNA-29c is less validated but has been described by us and others [12], [21]. These three markers can all be accurately measured by real-time PCR [9], [10], [12], an easily standardized and reproducible method. In addition, a three parameters score including mRNA and microRNA allows the analysis of different cellular mechanisms underlying the observed clinical evolution. Indeed, ZAP70 has been implicated in CLL cell migration [22], [23], a process that is particularly relevant in CLL biology [24]. LPL has linked alterations of lipid metabolism with CLL pathogenesis [25], [26]. MicroRNA-29c also reflects tumour burden, proliferation and disease aggressiveness [12], thus providing information about the stage of disease evolution at the time of diagnosis.

Compared to our preview study [12], the number of patients in the current study was increased (60 patients were added to the 110 patients initial cohort [12]) and their follow-up was updated. Surprisingly, microRNA-223 lost its prognostic power for OS prediction and was, therefore, excluded from the new qPCR score. It should be noted that the preview score including microRNA-223 remains significant in our 170 patient cohort, but the differences between subgroups was less pronounced (data not shown). Classification was, thus, improved (particularly during the first two years after diagnosis) and was simplified down to three subgroups: 0/3 (low risk), 1–2/3 (intermediate risk) and 3/3 (high risk). The idea is very simple: when all three factors are concordant in predicting patient evolution, the patient is classified into the low risk or high risk subgroup, but if at least one of the three factors is discordant the patient is included in the intermediate risk subgroup. After 105 months of follow-up, only 30% of the patients with a 0/3 score required treatment, whereas 65% of the 1–2/3 group and 100% of the 3/3 group required treatment. Furthermore, after 28 years of follow-up, patients with a 3/3 score had a 10-fold higher risk to require treatment and a 19-fold higher risk to die compared to patients with a 0/3 score. We also observed that this qPCR score permits the division of Binet stage A patients into three groups with a >210, 88 and 37 month median TFS, indicating that this score is an effective prognostic tool in early stage patients.

This qPCR score can clearly refine the prognostic power of old and new prognostic factors. Our statistical analyses reveal that this score allows the identification of patients with a worse prognosis or a less indolent evolution defined by the other recently applied prognostic factors. The qPCR score was able to refine CLL prognosis and produce a significant univariate HR even when we applied this score to IgVH mutational status-based subgroups (one of the most commonly used factors), as shown in Table 2. Therefore, this tool could be an interesting alternative for patient's lacking this information because of technical limitations. In addition, this score could divide patients in 3 subgroups while IgVH status only defined 2 subgroups. Similar results have been observed for proliferation (sCD23, β-2M, LDT), surface (CD38) or other RNA-based (CLLU1) markers. Cytogenetic abnormalities also represent a powerful prognosticator [17]. However, cytogenetic data were only available for 68% of our cohort. Therefore, Dohner's classification was adapted and patients were only separated in two groups. The distribution of patients with unfavourable cytogenetic abnormalities was 26% (10/38), 46% (25/55) and 72% (16/22) for subgroups 0/3, 1–2/3 and 3/3, respectively. This promising correlation has to be confirmed in a wider series, but our qPCR score has already defined different prognostic subgroups within cytogenetically favourable and unfavourable prognosis subgroups as stated in Figure 3. One of the most important observations is that the patient group with a score of 0/3 always had a TFS>210 months and an OS>330 months independently of the status of the other classical prognostic factors. An exception is made for Binet Stage B/C patients with a score of 0/3 who had a median TFS of 85 months. However, this value was calculated on only 2 cases. Finally, this score also displayed the best univariate HR for TFS and OS suggesting that, in our hands, this qPCR score is currently the most powerful molecular indicator for determining the prognosis of CLL patients.

One of the disadvantages of this score is the use of ROC curve analysis to fix the cut-off. Indeed, in comparison to our previous study, some cut-off remains quite stable (ZAP70 and microRNA-29c) while there is a substantial modification for LPL (which changes from 6 to 15) and microRNA-223 (from 82 to 138). For LPL, this modification only changes the status of 2 patients who became negative in the present study. However, the new calculated cut-off induced a status change of 24 patients for microRNA-223. This indicates that the microRNA-223 cut-off seems not to be stable and this can explain the lost of its prognostic value for OS prediction in the extended cohort. This cut-off instability is another argument for the exclusion of microRNA-223 from the present qPCR score. The determination of the optimal cut-off is a recurrent problem in all prognostic studies using continuous variables. The CD38 cut-off is a representative example. Cut-offs of 7 [27], 20 [28] and 30% [6] are all found in the literature. The use of ROC analysis is just a way to optimize concordance with TFS and OS. Optimally, cut-off points should be validated on a larger patient cohort. To be consistent in the present study, we also applied the same procedure for the determination of the other prognostic factor cut-offs.

The present study also has several strengths. First, the validity of this cohort for this kind of analysis is suggested by a consistent follow-up (median follow-up of 64 months with a maximum of 28 years) and the representative prognostic impact of other current prognostic factors (Table 1). Second, we performed our analyses on CD19^+^ purified samples. This score reflected thus an intrinsic characteristic of leukemic cells. A small number of studies used a cohort of highly purified CD19 cells. This aspect of the study could also be considered a negative point by others because of the so-called difficult and laborious purification and the need for special equipment. However, it should be noted that cell isolation using magnetic beads is an easy and rapid (less than 30 min) method. This technique requires only a magnet and columns, and the equipment is affordable enough to be obtained and installed in many routine laboratories. Furthermore, the availability of this qPCR score could be extended to non-specialized laboratories or those that are not qPCR-equipped because lysed cells can be easily sent to other labs at room temperature. Third, this model was confirmed in Binet stage A patients, the principal patient group in need of better prognostic tools. Fourth, this study was performed on samples collected at diagnosis, which is the most important time for prognostication. Fifth, our model was internally validated. Indeed, even if the 170 patient cohort was divided and reduced to 31 patients (as was the case for the LDT<1 year patients), our score was unaffected and remained a significant TFS predictor. However, before this score can be used for further applications, an external validation, ideally a prospective trial, should be performed.

For patients, prognostic information is also important in helping them to plan their lives. For clinician scientists, this score may provide insight into the biology of the disease because the exact roles of ZAP70, LPL and microRNA-29c are not yet completely elucidated. In conclusion, this qPCR score has powerful prognostic value and is easily, accurately determined, and reduces patient misclassification. This new prognostic tool could, thus, facilitate discussion between physicians and patients and help to identify patients who will require early therapy.

## Methods

### Ethics Statement

This study has been approved by the Bordet Institute Ethics Committee and conducted according to the principles expressed in the Declaration of Helsinki. All samples were collected after written informed consent.

### Patients, sample collection, and RNA extraction

This study analysed peripheral blood samples collected at the time of diagnosis from 170 CLL patients (with informed consent) with a typical CD19^+^CD5^+^CD23^+^ phenotype and blood leukocyte counts between 10×10^3^ and 250×10^3^ cells/µL. Table 1 summarizes other patient features. Treatment-free survival (TFS) and overall survival (OS) were calculated from the time of diagnosis until the date of first treatment and the date of death, respectively. All deaths were CLL-related. Peripheral blood mononuclear cells were isolated by density-gradient centrifugation over Linfosep (Biomedics, Madrid, Spain). B cells were purified with a CD19^+^ magnetic bead system (MidiMACS, Miltenyi Biotec, Bergish Gladbash, Germany), according to the manufacturer's instructions. Mean B cell purity was >99% as measured by flow cytometry. Total RNA was extracted from purified CD19^+^ cells in a single step using TriPure Isolation Reagent (Roche Applied Science, Vilvoorde, Belgium).

### Assessment of RNA-based prognostic factors

We measured ZAP70, LPL, and CLLU1 expression by qPCR as previously described [9], [10], [13]. ZAP70 and LPL were measured using Power SYBR® Green PCR Master Mix and CLLU1 (cDNA1 transcript) with TaqMan MGB probe and TaqMan® Universal PCR Master Mix (Applied Biosystems, Rotterdam, The Netherlands) because of the presence of different CLLU1 alternative transcripts. Expression levels were normalized against cyclophilin A (PPI) gene. MicroRNA expression was measured using the TaqMan microRNA quantitative PCR kit (Applied Biosystems) as previously described [12] and normalized with the endogenous control, RNU48 (purchased from Applied Biosystems). RNA-based makers were expressed as the fold-change of the target gene expression in the calibrator B lymphoid cell line Namalwa (for ZAP70, LPL, microRNA-29c and microRNA-223) or a pool of purified normal B cells (for CLLU1) because of the absence of CLLU1 in the cell line. All primers have been previously described [9], [10], [13]. The comparative ΔΔCt method was applied for data analysis.

### Assessment of other prognostic factors

CD38 expression was assessed by flow cytometry, sCD23 and β2-M by ELISA immunoassay, and IgVH gene mutational analysis was performed as previously described [5], [10], [14], [15]. LDT was assessed according to Montserrat et al. [16]. Classical cytogenetics by standard karyotype analysis and additional interphase FISH were performed to screen for most common aberrations using Chromoprobe Multiprobe® - CLL System (Cytocell, Amplitech, Compiegne, France). Patients were then classified according to Döhner's recommendations [17].

### Statistical analysis

We analysed ROC curves with GraphPad Prism 5.0 (Graph-Pad Software) to determine the ZAP70, LPL, CLLU1, miR-29c, miR-223, CD38, sCD23 and β2-M expression cut-off values that best distinguished mutated and unmutated cases. TFS and OS distributions were plotted using Kaplan-Meier estimates and were compared using the log-rank test or the Cox regression hazard ratio method for more than two subgroups. Univariate Cox regression analysis evaluated the effects of the different prognostic variables on TFS and/or OS. All tests were two-sided. An effect was considered to be statistically significant at *P<0.05*. All analyses were performed with SPSS 15.0 software.
